# Evaluation of artificial intelligence-based electrocardiogram analysis tools in patients with hypertrophic cardiomyopathy

**DOI:** 10.1093/ehjdh/ztag026

**Published:** 2026-02-26

**Authors:** Gamze Babur Guler, Arda Guler, Ozgur Surgit, Irem Turkmen, Sezgin Atmaca, Hasan Sahin, Dilara Pay, Muayad Almasri, Gizemnur Coskun, Utku Yartasi, Dogukan Salduz, Busra Kuru Gorgulu, Sinem Aydin, Nail Guven Serbest, Aysel Turkvatan Cansever, Ibrahim Halil Tanboga

**Affiliations:** Mehmet Akif Ersoy Thoracic and Cardiovascular Surgery Training and Research Hospital, Cardiology, Istanbul, Turkey; Mehmet Akif Ersoy Thoracic and Cardiovascular Surgery Training and Research Hospital, Cardiology, Istanbul, Turkey; Mehmet Akif Ersoy Thoracic and Cardiovascular Surgery Training and Research Hospital, Cardiology, Istanbul, Turkey; Mehmet Akif Ersoy Thoracic and Cardiovascular Surgery Training and Research Hospital, Cardiology, Istanbul, Turkey; Mehmet Akif Ersoy Thoracic and Cardiovascular Surgery Training and Research Hospital, Cardiology, Istanbul, Turkey; Mehmet Akif Ersoy Thoracic and Cardiovascular Surgery Training and Research Hospital, Cardiology, Istanbul, Turkey; Mehmet Akif Ersoy Thoracic and Cardiovascular Surgery Training and Research Hospital, Cardiology, Istanbul, Turkey; Mehmet Akif Ersoy Thoracic and Cardiovascular Surgery Training and Research Hospital, Cardiology, Istanbul, Turkey; Mehmet Akif Ersoy Thoracic and Cardiovascular Surgery Training and Research Hospital, Cardiology, Istanbul, Turkey; Mehmet Akif Ersoy Thoracic and Cardiovascular Surgery Training and Research Hospital, Cardiology, Istanbul, Turkey; Mehmet Akif Ersoy Thoracic and Cardiovascular Surgery Training and Research Hospital, Cardiology, Istanbul, Turkey; Mehmet Akif Ersoy Thoracic and Cardiovascular Surgery Training and Research Hospital, Cardiology, Istanbul, Turkey; Mehmet Akif Ersoy Thoracic and Cardiovascular Surgery Training and Research Hospital, Radiology, Istanbul, Turkey; Mehmet Akif Ersoy Thoracic and Cardiovascular Surgery Training and Research Hospital, Cardiology, Istanbul, Turkey; Mehmet Akif Ersoy Thoracic and Cardiovascular Surgery Training and Research Hospital, Radiology, Istanbul, Turkey; Istanbul Nisantasi University Medical School, Cardiology & Biostatistics and Bioinformatics, Istanbul, Turkey

**Keywords:** Artificial intelligence, Electrocardiogram, Hypertrophic cardiomyopathy, Diagnostic accuracy

## Abstract

**Aims:**

Artificial intelligence (AI)-based electrocardiogram (ECG) analysis tools have shown promise in detecting various cardiac conditions. However, their performance in specific patient populations, such as those with hypertrophic cardiomyopathy (HCM), remains incompletely characterized. To evaluate the performance of three AI-based ECG analysis tools in patients with confirmed HCM: (1) a tool calculating HCM probability, (2) a tool calculating structural heart disease (SHD) probability, and (3) a tool providing ECG-based diagnoses across multiple categories.

**Methods and results:**

We analysed digitized 12-lead ECGs from patients with confirmed HCM (*n* = 681) using three AI tools. We assessed the distribution of AI-calculated probabilities and their associations with clinical parameters and evaluated agreement between AI-based and manually assigned ECG diagnoses using Cohen’s kappa. Despite all patients having confirmed HCM, the AI-calculated HCM probabilities showed a relatively uniform distribution [median 38.8% (IQR: 12.8–63.4%)], with only 41.2% and 12.5% of patients receiving a probability score >50% and >75%. HCM probabilities were significantly higher in patients with abnormal vs. normal ECGs (*P* < 0.001) and correlated with markers of disease severity. SHD probabilities were generally higher [median 51.4% (IQR: 28.7–74.5%)], with 51.2% and 25% of patients receiving scores >50% and >75%.

**Conclusion:**

AI-based ECG analysis tools demonstrated modest performance in our HCM cohort. These findings highlight the challenges of applying AI tools developed in general populations to specific disease cohorts and underscore the need for disease-specific validation before clinical implementation.

## Graphical Abstract

**Graphical Abstract:**
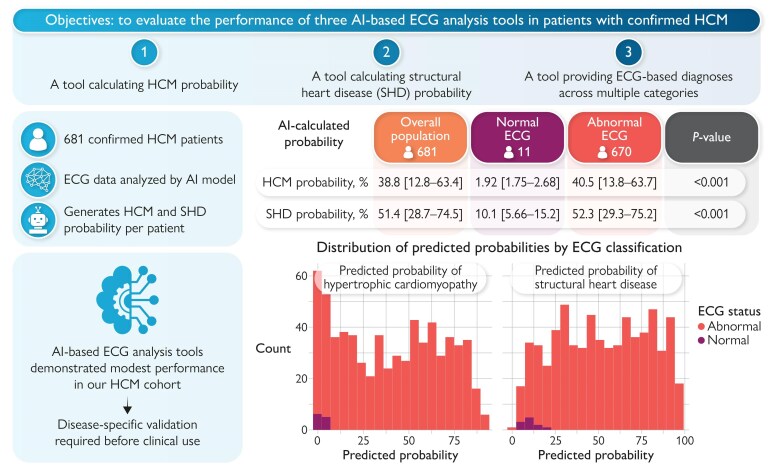

## Introduction

Hypertrophic cardiomyopathy (HCM) is a common inherited cardiac disorder characterized by unexplained left ventricular hypertrophy, affecting approximately 1 in 500 individuals in the general population.^1^ It represents a significant cause of sudden cardiac death in young adults and athletes, while also contributing to heart failure and stroke in older patients. The 12-lead electrocardiogram (ECG) remains a cornerstone in the initial evaluation of patients with suspected or established HCM.^2^ ECG abnormalities are present in up to 90% of HCM patients, with common findings including left ventricular hypertrophy, ST-segment and T-wave abnormalities, pathological Q-waves, and conduction disturbances.^2^ Despite its widespread availability and relatively low cost, the interpretation of ECGs requires considerable expertise, and inter-observer variability can impact diagnostic accuracy and clinical decision-making.

In recent years, artificial intelligence (AI) has emerged as a promising approach to enhance ECG interpretation.^3^ AI-based diagnostic tools have demonstrated remarkable capabilities in analysing 12-lead ECGs for various cardiac conditions, potentially improving diagnostic accuracy, efficiency, and accessibility of cardiac care.^4,5^ Several AI algorithms have been developed specifically for ECG interpretation, including tools for detecting HCM, identifying structural heart disease (SHD), and providing comprehensive ECG-based diagnoses across multiple categories.^6,7^

These AI tools have typically been developed and validated in diverse populations, often including both healthy individuals and those with various cardiac conditions. However, their performance in specific patient populations, particularly those with established diagnoses such as HCM, remains incompletely characterized. Testing these AI tools in a population exclusively comprising patients with confirmed HCM offers a unique opportunity to evaluate their diagnostic performance, limitations, and potential clinical utility in this specific context.^7,8^

The aim of this study was to evaluate the performance of three distinct AI-based ECG analysis tools in a well-characterized cohort of patients with established HCM. Designed as a post-validation, disease-focused evaluation, the study sought to assess the distribution of AI-calculated HCM and SHD probability scores across the clinical spectrum of HCM, evaluate the agreement between AI-based outputs and expert ECG interpretations across multiple diagnostic categories, and analyse the associations between ECG abnormalities and disease.

## Methods

### Study population

This study included patients with a confirmed diagnosis of HCM from our institutional database. All patients met established diagnostic criteria for HCM, defined as unexplained left ventricular hypertrophy with maximal wall thickness ≥15 mm (or ≥13 mm in first-degree relatives of HCM patients) in the absence of another cardiac or systemic disease capable of producing such magnitude of hypertrophy.^9^ The study cohort comprised patients with HCM for whom detailed demographic, clinical, laboratory, echocardiographic, cardiac magnetic resonance (CMR), genetic, and electrocardiographic data were collected as part of routine clinical care. Genetic testing was offered to all patients, and performed in those who provided consent, using next-generation sequencing panels that included key sarcomeric and related genes known to be associated with HCM, such as MYH7, MYBPC3, and TNNT2. A result was considered positive if a pathogenic or likely pathogenic variant was identified. In cases where a variant of uncertain significance (VUS) was detected—particularly in one of the cores HCM-related genes and if supported by family history—segregation analysis was performed in first-degree relatives. Variant interpretation was supported by multiple databases including ClinVar, gnomAD, HGMD, and in silico prediction tools (e.g. CADD, SIFT, MutationTaster).

The study was approved by the institutional ethics committee and conducted in accordance with the principles of the Declaration of Helsinki.

### Electrocardiographic assessment

During routine clinical evaluation, standard 12-lead ECGs were obtained from all patients. The ECGs were recorded using standard digital ECG equipment at a paper speed of 25 mm/s and an amplitude of 10 mm/mV. All patients’ ECGs were digitized and stored as images in our hospital's data system. For this study, all ECGs were uploaded for analysis by three AI tools. All ECGs were initially interpreted by experienced cardiologists as part of routine clinical care, with findings documented in the electronic medical record.

An ECG, manually, was classified as abnormal if any of the following criteria were met:—atrial fibrillation (paroxysmal or persistent)—presence of AV block—bundle branch block (including LBBB, RBBB, or indeterminate)—pathological Q-waves—T-wave inversion, deep T-waves, or giant T-waves—biphasic T-waves—ST-segment depression or elevation—Sokolow–Lyon index ≥ 35 mm—low voltage—QTc interval > 460 ms—QRS duration > 120 ms.

### Artificial intelligence tools

Three distinct AI-based ECG analysis tools were evaluated in this study:

1. **HCM Probability Tool**: This AI algorithm was specifically developed to calculate the probability of HCM based on 12-lead ECG analysis. The tool was developed by researchers at Yale University and utilizes deep learning techniques to analyse ECG patterns associated with HCM. The algorithm was trained on a large dataset of ECGs from both HCM patients and controls and has been previously validated for HCM detection in general populations (https://www.cards-lab.org/ecgvision-hcm).^6^
2. **Structural Heart Disease (SHD) Probability Tool**: This ensemble deep learning algorithm (PRESENT SHD) was designed to screen for SHD using electrocardiographic images. Developed by Dhingra *et al*., this tool analyses 12-lead ECGs to calculate the probability of underlying structural heart abnormalities. While not specific to HCM, this tool provides a broader assessment of cardiac structural abnormalities that might be present in HCM patients (https://www.cards-lab.org/present-shd).^7^
3. **ECG Diagnostic Categorization Tool**: This automated multilabel diagnosis system, developed by Sangha *et al*., provides diagnostic interpretations across multiple categories based on ECG analysis. The tool was trained on over 2.2 million 12-lead ECGs and can identify six physician-defined clinical labels spanning rhythm and conduction disorders. This system allows for comprehensive ECG interpretation beyond single-disease probability assessment (https://www.cards-lab.org/ecgdx).^3^

### Application of AI tools

All three AI tools were applied to the digitized 12-lead ECG from our HCM cohort. For each patient, the following outputs were recorded:

1. The calculated probability of HCM (expressed as a percentage) from the HCM Probability Tool.
2. The calculated probability of SHD (expressed as a percentage) from the SHD Probability Tool.
3. The diagnostic categories identified by the ECG Diagnostic Categorization Tool.

### Data validation process

To assess the performance of the AI tools, we conducted a manual validation process comparing the AI-based ECG diagnoses with our clinical assessments. For the HCM and SHD probability tools, we analysed the distribution of calculated probabilities across the entire cohort, as well as in relation to various clinical, echocardiographic, and ECG parameters.

For the ECG Diagnostic Categorization Tool, we compared the AI-assigned diagnostic categories with the manually determined ECG diagnoses from our clinical database. This comparison focused on key diagnostic categories including atrial fibrillation, atrioventricular block, bundle branch blocks, and other rhythm and conduction abnormalities. Agreement between AI and manual diagnoses was quantified using appropriate statistical measures as described in the statistical analysis section.

## Statistical analysis

All statistical analyses were performed using R (version 4.1.0, R Foundation for Statistical Computing, Vienna, Austria). Continuous variables were summarized as mean ± standard deviation and median [interquartile range] to account for potential non-normal distributions. Categorical variables were presented as counts and percentages. The distributions of AI-calculated probabilities (HCM and SHD) were visualized using histograms and box plots, stratified by ECG classification (normal vs. abnormal). A two-sided *P*-value <0.05 was considered statistically significant for all analyses.

Differences in AI-calculated probabilities between groups (e.g. normal vs. abnormal ECG) were assessed using the Wilcoxon rank-sum test due to non-normal distributions of the probability values. For categorical analyses, AI-calculated probabilities were categorized into quartiles (<25%, 25–50%, 50–75%, and >75%) to facilitate clinical interpretation and comparison across patient subgroups. Relationships between continuous variables (e.g. AI-calculated probabilities and clinical/echocardiographic parameters) were evaluated using Pearson correlation coefficients, with scatter plots and regression lines to visualize these relationships. For categorical variables, differences in AI-calculated probabilities across categories were assessed using the Wilcoxon rank-sum test (for binary variables) or the Kruskal–Wallis test (for variables with more than two categories).

To evaluate the agreement between AI-based and manually assigned ECG diagnoses, we calculated Cohen’s kappa coefficients. The kappa statistic measures inter-rater agreement for categorical items, accounting for the possibility of agreement occurring by chance. Kappa values were interpreted as follows: <0 (no agreement), 0–0.20 (slight agreement), 0.21–0.40 (fair agreement), 0.41–0.60 (moderate agreement), 0.61–0.80 (substantial agreement), and 0.81–1.00 (almost perfect agreement). For each diagnostic category, we also calculated the percentage agreement, sensitivity, specificity, positive predictive value, and negative predictive value of the AI-based diagnoses compared with the manual diagnoses.

## Results

### Study population characteristics

The study cohort consisted of 681 patients with confirmed HCM. Demographic and clinical characteristics of the study population are summarized in *Table 1*. The mean age was 52.8 ± 13.4 years, with a male predominance (68%). The cohort included patients with various HCM subtypes: obstructive (52%), non-obstructive (39%), and apical (9%). Family history of HCM was present in 16% of patients, and family history of sudden cardiac death in 12%. The mean SCD risk score was 2.99 ± 2.26.

**Table 1:** Baseline clinical characteristics of the study population

Characteristic	Value
**Demographics**	
Age, years	52.8 ± 13.4
Male gender, *n* (%)	463 (68%)
**HCM characteristics**	
HCM type	
− Obstructive, *n* (%)	353 (52%)
− Non-obstructive, *n* (%)	263 (39%)
− Apical, *n* (%)	61 (9%)
Family history of HCM, *n* (%)	107 (16%)
Family history of SCD, *n* (%)	80 (12%)
SCD risk score	2.99 ± 2.26
**Previous histories**	
Hypertension, *n* (%)	347 (52%)
Diabetes, *n* (%)	98 (15%)
Coronary artery disease, *n* (%)	186 (28%)
ICD implantation, *n* (%)	75 (11%)
Myectomy history, *n* (%)	17 (2.5%)
Alcohol ablation history, *n* (%)	37 (5.5%)
**Laboratory parameters**	
NT-proBNP, pg/mL	1283 ± 2398
Creatinine, mg/dL	0.95 ± 0.43
**Echocardiographic parameters**	
LVEF, %	59.6 ± 7.8
Maximal wall thickness, mm	18.6 ± 4.1
Left atrial diameter, mm	41.9 ± 6.6
Moderate to severe MR, *n* (%)	124 (20%)
**CMR parameters**	
CMR-LVEF, %	66.1 ± 8.6
Late gadolinium enhancement, *n* (%)	489 (86%)
Diffuse LGE, *n* (%)	141 (25%)

Values are presented as mean ± standard deviation, median [interquartile range], or *n* (%).

HCM, hypertrophic cardiomyopathy; SCD, sudden cardiac death; ICD, implantable cardioverter-defibrillator; NT-proBNP, N-terminal pro-B-type natriuretic peptide; LVEF, left ventricular ejection fraction; IVS, interventricular septum; MR, mitral regurgitation; CMR, cardiac magnetic resonance; LGE, late gadolinium enhancement; ECG, electrocardiogram; AV, atrioventricular.

Common comorbidities included hypertension (52%), coronary artery disease (28%), and diabetes (15%). Regarding interventions, 11% had undergone ICD implantation, 2.5% had a history of myectomy, and 5.5% had undergone alcohol septal ablation. The mean maximal wall thickness was 18.6 ± 4.1 mm, and the mean left atrial diameter was 41.9 ± 6.6 mm. Cardiac MRI was present in 84% of patients and late gadolinium enhancement was present in 86% of patients, with diffuse LGE in 25%.

We have the genetic test results for 211 patients (31% of the overall cohort). Among those tested, 41% had a positive result, 37% were negative, and 22% carried a VUS. The most frequently identified pathogenic or likely pathogenic mutations were in MYBPC3 (39% of positive cases) and MYH7 (26%), followed by MYL3 and TNNI3.

### Distribution of AI-calculated HCM probabilities

**Figure 1:**
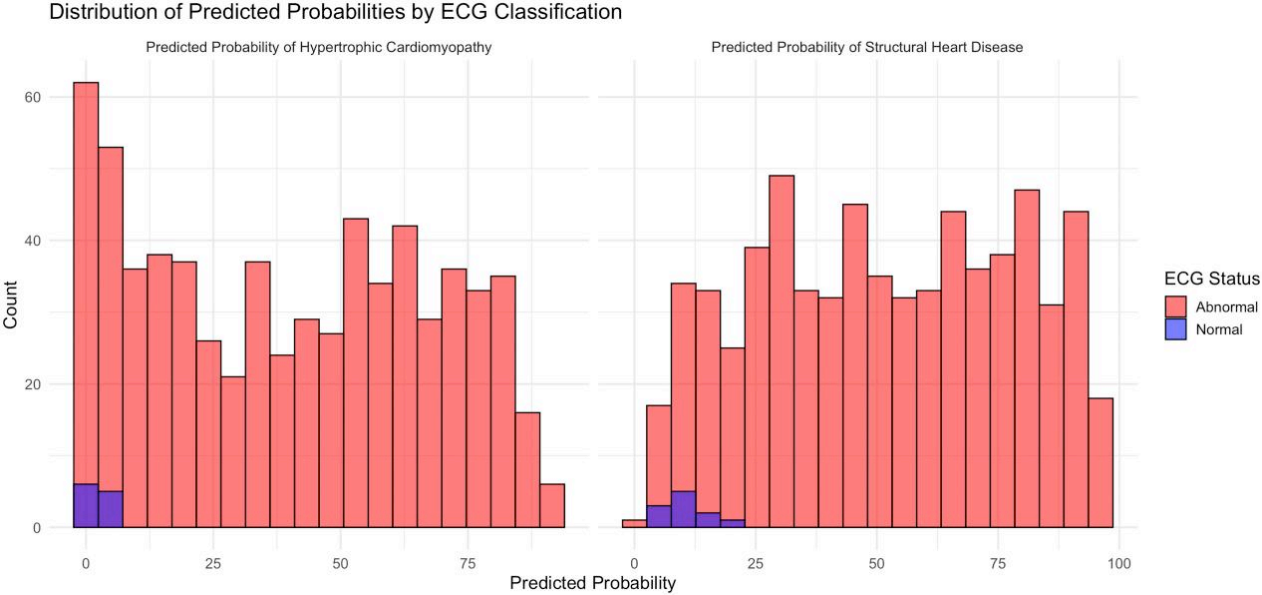
Distribution of AI-calculated HCM and structural heart disease probabilities in patients with confirmed HCM histogram showing the distribution of AI-calculated probabilities of hypertrophic cardiomyopathy (HCM) and structural heart disease (SHD) probabilities across the entire cohort.

The distribution of AI-calculated HCM probabilities across the entire cohort is presented in *Figure 1*. Despite all patients having confirmed HCM, the AI-calculated HCM probabilities showed a relatively uniform distribution [median 38.8% (IQR: 12.8–63.4%)], with only 41.2% and 12.5% of patients receiving a probability score >50% and >75%.

**Figure 2:**
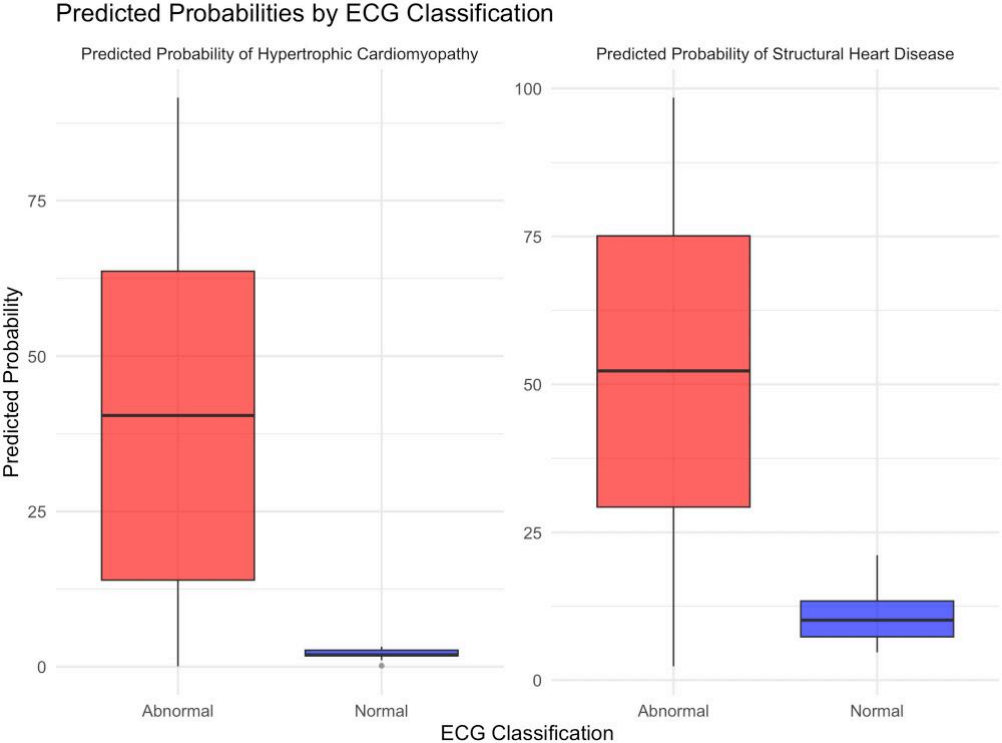
HCM and SHD probabilities stratified by ECG classification box plots comparing AI-calculated HCM and SHD probabilities between patients with normal vs. abnormal ECGs.

**Figure 3:**
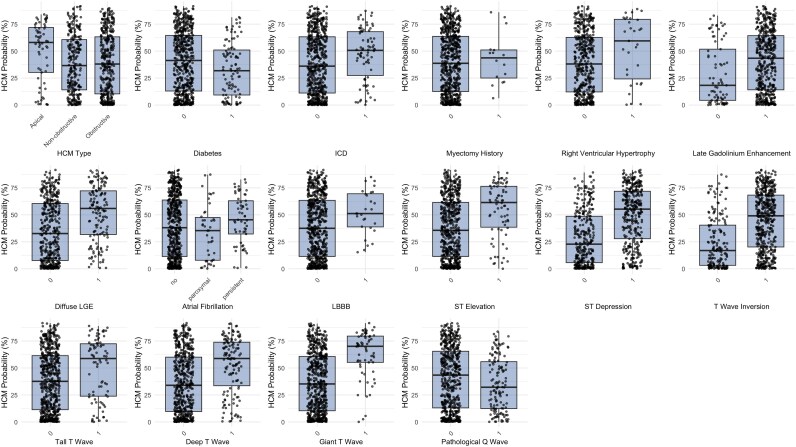
Correlation between HCM probability and clinical, echocardiographic, and ECG parameters.

ECG characteristics are shown in *Table 2*. When stratified by ECG classification (normal vs. abnormal), patients with abnormal ECGs demonstrated significantly higher AI-calculated HCM probabilities compared with those with normal ECGs [median 40.5% (IQR: 13.8–63.7%) vs. 1.92% (IQR: 1.75–2.68%), *P* < 0.001] (*Table 3*). Analysis of HCM probabilities across different clinical subgroups revealed several significant associations (*Tables 4* and *Figure 2*). Patients with more severe phenotypic expressions of HCM, including greater maximal wall thickness (*r* = 0.30, *P* < 0.001), higher NT-proBNP levels (*r* = 0.41, *P* < 0.001), and larger left atrial diameter (*r* = 0.16, *P* < 0.001), demonstrated higher AI-calculated HCM probabilities. Additionally, patients with specific ECG abnormalities, including T-wave inversion [49.1% (20.2–68.3%) vs. 16.9% (3.2–40.4%), *P* < 0.001], ST depression [55.3% (27.9–71.9%) vs. 22.9% (5.7–48.7%), *P* < 0.001], and ST elevation [61.4% (38.6–76.5%) vs. 35.6% (11.5–61.5%), *P* < 0.001], had significantly higher HCM probabilities compared with those without these findings. In addition, HCM probabilities varied significantly by HCM type (*Table 5* and *Figure 3*), with apical variant showing the highest probabilities [58.0% (30.3–71.9%)], followed by obstructive [38.0% (10.4–63.2%)] and non-obstructive [36.6% (14.1–60.6%)] variants (*P* = 0.016). Patients with ICD implantation had higher HCM probabilities [50.7% (27.5–68.0%) vs. 36.1% (11.2–63.3%), *P* = 0.011], while diabetic patients had lower probabilities [31.8% (9.3–51.0%) vs. 41.3% (13.0–64.5%), *P* = 0.010].

**Table 2:** Baseline ECG characteristics of the study population

Characteristic	Value
Atrial fibrillation, *n* (%)	34 (24.8%)
Atrioventricular block, *n* (%)	21 (15.3%)
Heart rate, beat/min	72.3 ± 13.1
PR interval, ms	176 ± 32.4
QRS duration, ms	105 ± 24.3
QTc interval, ms	453 ± 36.5
P-wave axis, ^o^	47.4 ± 22.0
R-wave axis, ^o^	15.4 ± 42.0
T-wave axis, ^o^	109 ± 75.0
T-wave inversion, *n* (%)	82 (59.9%)
Pathological Q-waves, *n* (%)	43 (31.4%)
Left bundle branch block, *n* (%)	18 (13.1%)
Right bundle branch block, *n* (%)	12 (8.8%)
ST segment elevation, *n* (%)	74 (11%)
ST segment depression, *n* (%)	356 (53%)
T-wave inversion, *n* (%)	502 (74%)
Biphasic T-wave, *n* (%)	82 (12%)
Tall T-wave, *n* (%)	82 (12%)
Deep T-wave, *n* (%)	138 (20%)
Giant deep T-wave, *n* (%)	66 (9.7%)
Sokolow–Lyon index	37.51 ± 12.69
Low voltage, *n* (%)	18 (2.7%)
Tp/Te interval	71.7 ± 28.7

**Table 3:** AI-calculated probabilities stratified by ECG classification

AI-calculated probability	Overall population (*n* = 681)	Normal ECG (*n* = 11)	Abnormal ECG (*n* = 670)	*P*-value
HCM probability, %	38.8 [12.8–63.4]	1.92 [1.75–2.68]	40.5 [13.8–63.7]	<0.001
SHD probability, %	51.4 [28.7–74.5]	10.1 [5.66–15.2]	52.3 [29.3–75.2]	<0.001

Values are presented as median [interquartile range].

HCM, hypertrophic cardiomyopathy; SHD, structural heart disease; ECG, electrocardiogram. *P*-values calculated using Wilcoxon rank-sum test.

**Table 4:** Correlations between HCM probabilities and clinical variables

Continuous variable	Correlation coefficient (*r*)	*P*-value
Maximal wall thickness	0.30	<0.001
Left atrial diameter	0.16	<0.001
NT-proBNP (log-transformed)	0.41	<0.001
QRS duration	0.26	<0.001
QTc interval	0.25	<0.001
SCD risk score	0.20	<0.001
Interventricular septum thickness	0.27	<0.001
Posterior wall thickness	0.22	<0.001
Sokolow index	0.39	<0.001
T-axis	0.48	<0.001
Tp/Te	0.13	<0.001

HCM, hypertrophic cardiomyopathy; NT-proBNP, N-terminal pro-B-type natriuretic peptide; SCD, sudden cardiac death.

**Table 5:** Associations between HCM probabilities and clinical variables

Variable	Categories	Median [IQR]	*P*-value
HCM type	Apical, Non-obstructive, Obstructive	58.0 [30.3–71.9], 36.6 [14.1–60.6], 38.0 [10.4–63.2]	0.016
Diabetes	Absent vs. Present	41.3 [13.0–64.5] vs. 31.8 [9.3–51.0]	0.010
ICD	Absent vs. Present	36.1 [11.2–63.3] vs. 50.7 [27.5–68.0]	0.011
Myectomy history	Absent vs. Present	38.7 [12.6–63.7] vs. 43.7 [25.0–51.1]	0.568
Right ventricular hypertrophy	Absent vs. Present	38.1 [12.2–62.7] vs. 59.5 [24.2–79.4]	0.011
Late gadolinium enhancement	Absent vs. Present	18.3 [4.4–51.8] vs. 43.4 [14.2–64.3]	<0.001
Diffuse LGE	Absent vs. Present	32.7 [7.7–60.5] vs. 55.9 [31.9–72.2]	<0.001
Atrial fibrillation	no, paroxymal, persistent	38.1 [11.4–63.7], 35.4 [7.9–47.6], 45.4 [32.3–63.0]	0.091
LBBB	Absent vs. Present	37.5 [11.5–63.4] vs. 51.2 [38.8–69.6]	0.013
ST elevation	Absent vs. Present	35.6 [11.5–61.5] vs. 61.4 [38.6–76.5]	<0.001
ST depression	Absent vs. Present	22.9 [5.7–48.7] vs. 55.3 [27.9–71.9]	<0.001
T-wave inversion	Absent vs. Present	16.9 [3.2–40.4] vs. 49.1 [20.2–68.3]	<0.001
Tall T-wave	Absent vs. Present	37.6 [11.3–61.4] vs. 58.8 [23.9–72.4]	<0.001
Deep T-wave	Absent vs. Present	34.0 [9.8–60.0] vs. 58.8 [33.6–73.9]	<0.001
Giant T-wave	Absent vs. Present	35.2 [10.4–60.7] vs. 70.1 [55.2–79.5]	<0.001
Pathological Q-wave	Absent vs. Present	43.4 [13.0–65.5] vs. 32.2 [12.4–55.9]	0.062

HCM, hypertrophic cardiomyopathy; ICD, implantable cardioverter defibrillator; LGE, late gadolinium enhancement; LBBB, left bundle branch block.

### Distribution of AI-calculated structural heart disease probabilities

**Figure 4:**
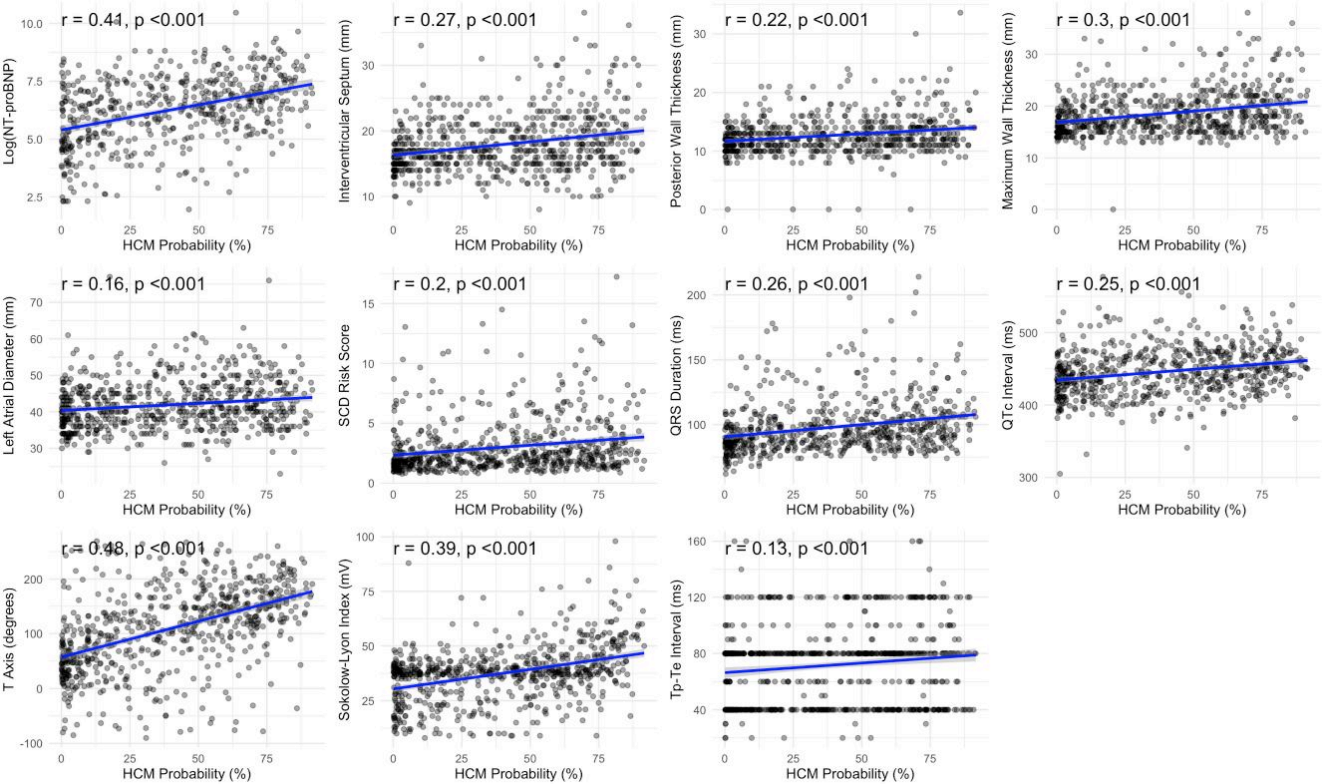
HCM probability across various clinical and imaging subgroups.

**Figure 5:**
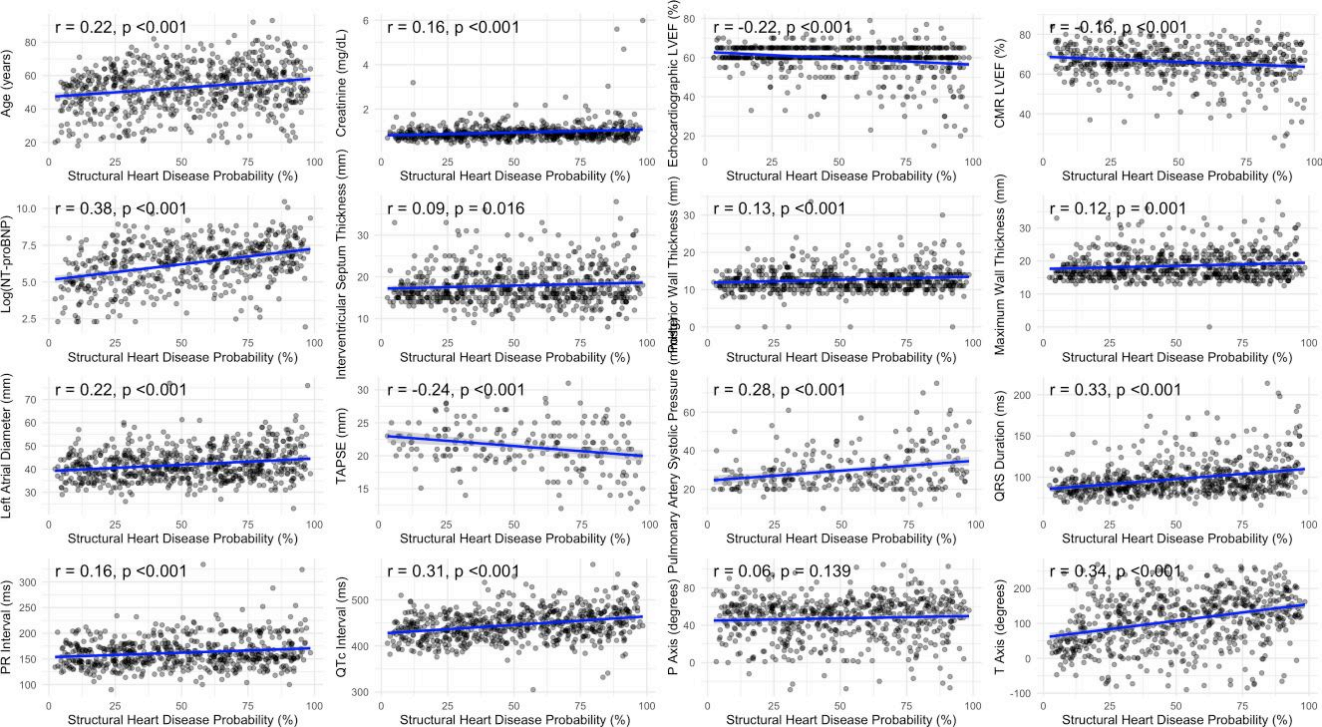
Correlation between structural heart disease probability and clinical, imaging, and ECG parameters.

The AI-calculated SHD probabilities showed a different distribution pattern compared with HCM probabilities (*Figure 4*). SHD probabilities were generally higher than the median HCM probability in this confirmed HCM population [median 51.4% (IQR: 28.7–74.5%)], with 51.2% and 25% of patients receiving scores >50% and >75%. Similar to HCM probabilities, SHD probabilities were significantly higher in patients with abnormal ECGs compared with those with normal ECGs [median 52.3% (IQR: 29.3–75.2%) vs. 10.1% (IQR: 5.66–15.2%), *P* < 0.001]. Correlation analyses revealed significant associations between SHD probabilities and various clinical and imaging parameters (*Table 6*, *Figure 5*). Notably, SHD probabilities showed correlations with NT-proBNP (log-transformed) (*r* = 0.38, *P* < 0.001), QTc interval (*r* = 0.31, *P* < 0.001), QRS duration (*r* = 0.33, *P* < 0.001), T-axis (*r* = 0.34, *P* < 0.001), age (*r* = 0.22, *P* < 0.001), and left atrial diameter (*r* = 0.22, *P* < 0.001). In addition, SHD probabilities showed negative correlations with left ventricular ejection fraction (LVEF) by echocardiography (*r* = −0.22, *P* < 0.001), LVEF by CMR (*r* = −0.16, *P* < 0.001), and tricuspid annular plane systolic excursion (*r* = −0.24, *P* < 0.001), suggesting higher probabilities in patients with reduced cardiac function.

**Table 6:** Correlations between SHD probabilities and clinical variables

Continuous variable	Correlation coefficient (*r*)	*P*-value
Age	0.22	<0.001
Left atrial diameter	0.22	<0.001
Interventricular septum thickness	0.09	0.016
Posterior wall thickness	0.13	<0.001
Maximum wall thickness	0.12	0.001
PR interval	0.16	<0.001
QTc interval	0.31	<0.001
NT-proBNP (log-transformed)	0.38	<0.001
Creatinine	0.16	<0.001
LVEF (by echocardiography)	−0.22	<0.001
LVEF (by CMR)	−0.16	<0.001
QRS duration	0.33	<0.001
P-axis	0.06	0.139
T-axis	0.34	<0.001
TAPSE	−0.24	<0.001
PASP	0.28	<0.001

SHD, structural heart disease; NT-proBNP, N-terminal pro-B-type natriuretic peptide; LVEF, left ventricular ejection fraction; CMR, cardiac magnetic resonance imaging; TAPSE, tricuspid annular plane systolic excursion; PASP, pulmonary artery systolic pressure.

**Figure 6:**
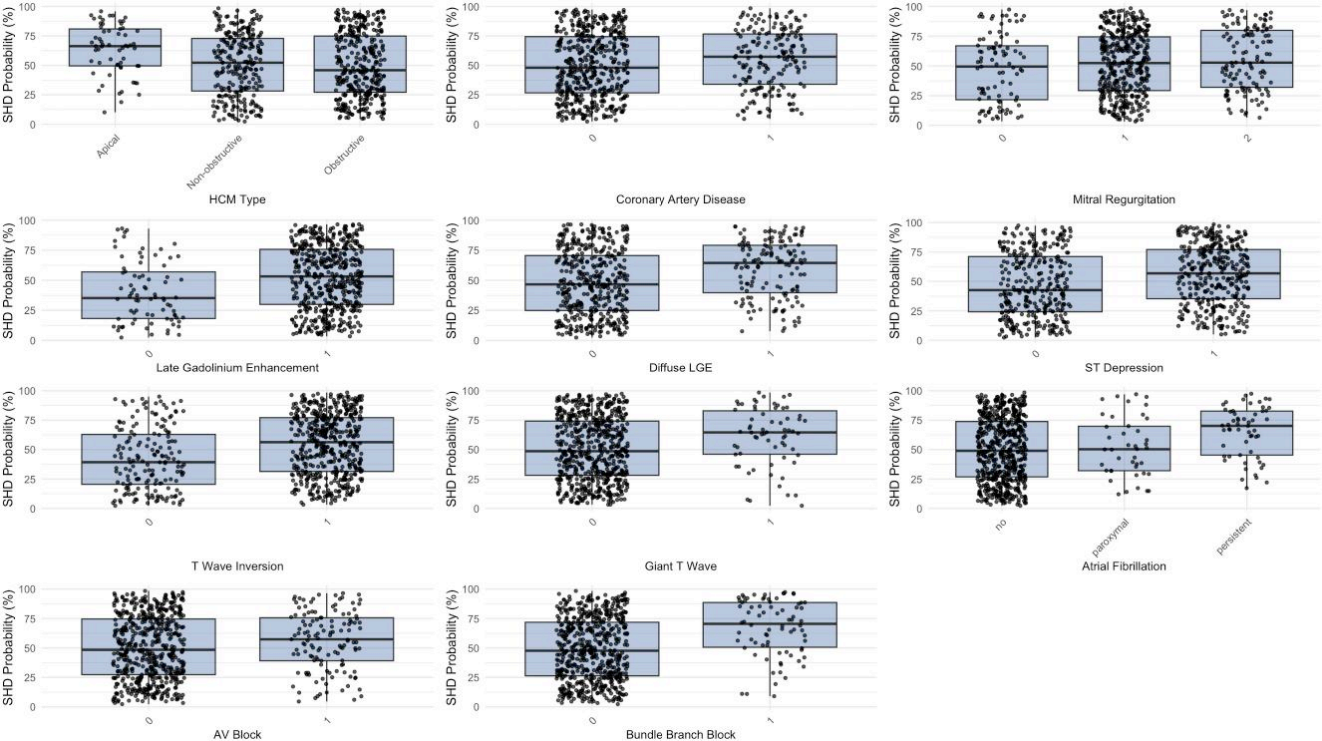
Structural heart disease probability across different structural and electrocardiographic phenotypes.

As shown in *Table 7* and *Figure 6*, SHD probabilities varied significantly across several clinical subgroups. Patients with apical HCM had higher median probabilities compared with obstructive types [66.3 (49.6–80.9) vs. 45.9 (27.2–74.9), *P* = 0.001]. The presence of coronary artery disease was also associated with increased probabilities [57.4 (34.0–76.7) vs. 48.0 (26.7–74.4), *P* = 0.022]. Similarly, late gadolinium enhancement and diffuse LGE were linked to markedly higher values [53.2 (29.9–75.7) vs. 35.2 (18.2–57.0) and 64.4 (39.6–79.1) vs. 46.6 (24.8–70.5), both *P* < 0.001]. ECG abnormalities such as ST depression, T-wave inversion, and giant T waves showed consistent associations, with median probabilities ranging from 56.3 to 64.6 compared with 39.3 to 48.6 in those without these changes (*P* ≤ 0.002). Atrial fibrillation, AV block, and bundle branch block also demonstrated significant associations, particularly persistent AF [70.2 (45.3–82.7), *P* < 0.001] and BBB [70.5 (50.6–88.6), *P* < 0.001].

**Table 7:** Associations between SHD probabilities and clinical variables

Variable	Categories	Median [IQR]	*P*-value
HCM type	Apical, Non-obstructive, Obstructive	66.3 [49.6–80.9], 52.3 [28.2–72.9], 45.9 [27.2–74.9]	0.001
Coronary artery disease	Absent vs. Present	48.0 [26.7–74.4] vs. 57.4 [34.0–76.7]	0.022
Mitral regurgitation	None, mild, moderate to severe	49.5 [21.6–66.9], 52.4 [29.3–74.5], 52.8 [32.0–79.9]	0.092
Late gadolinium enhancement	Absent vs. Present	35.2 [18.2–57.0] vs. 53.2 [29.9–75.7]	<0.001
Diffuse LGE	Absent vs. Present	46.6 [24.8–70.5] vs. 64.4 [39.6–79.1]	<0.001
ST depression	Absent vs. Present	42.6 [24.2–71.1] vs. 56.9 [35.4–77.1]	<0.001
T-wave inversion	Absent vs. Present	39.3 [20.5–62.9] vs. 56.3 [31.5–77.1]	<0.001
Giant T-wave	Absent vs. Present	48.6 [28.2–74.2] vs. 64.6 [46.1–83.0]	0.002
Atrial fibrillation	no, paroxymal, persistent	49.0 [26.8–73.8], 50.3 [32.1–69.8], 70.2 [45.3–82.7]	<0.001
AV block	Absent vs. Present	48.5 [27.3–74.6] vs. 57.4 [39.1–75.7]	0.034
Bundle branch block	Absent vs. Present	47.7 [26.4–71.9] vs. 70.5 [50.6–88.6]	<0.001

SHD, structural heart disease; HCM, hypertropic cardiomyopathy; LGE, late gadolinium enhancement; AV, atrioventricular.

### ECG diagnosis: manual vs. AI

The ECG characteristics of the study population are presented in *Table 2*. Atrial fibrillation was present in 24.8% of patients, and first-degree atrioventricular block in 15.3%. The mean heart rate was 72.3 ± 13.1 beats/min, with mean PR interval of 176 ± 32.4 ms, QRS duration of 105 ± 24.3 ms, and QTc interval of 453 ± 36.5 ms. Common ECG abnormalities included T-wave inversion (74%), ST segment depression (53%), pathological Q-waves (31.4%), left bundle branch block (13.1%), and right bundle branch block (8.8%).

**Figure 7:**
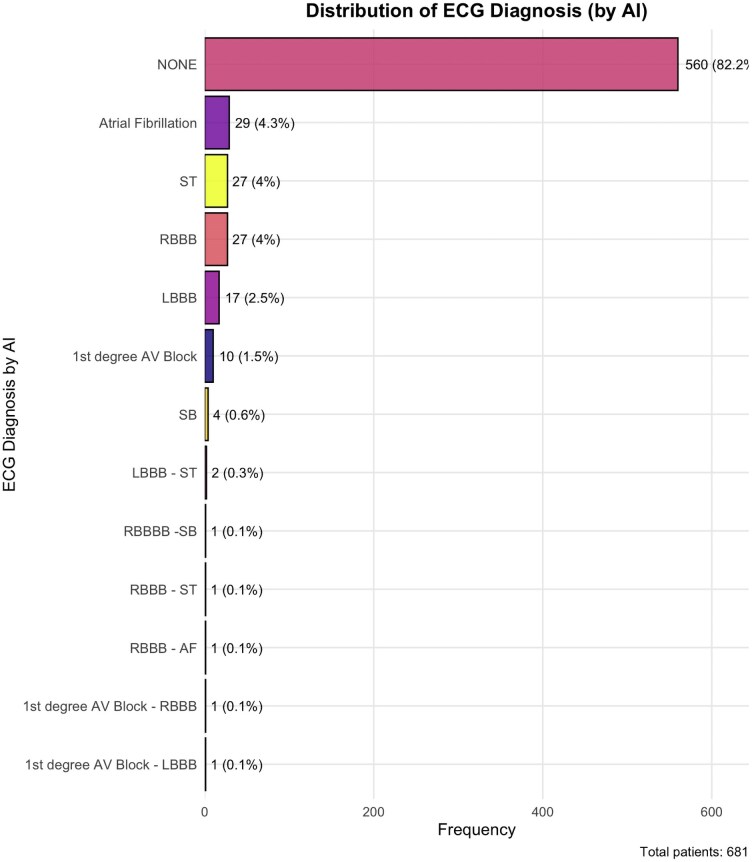
Distribution of ECG diagnoses derived from AI-based analysis. Bar plot displays the frequency and percentage of different ECG diagnoses in the study cohort.

Distribution of AI-based ECG diagnoses is shown in *Figure 7*. Among 681 patients, the majority (82.2%) received no abnormal ECG diagnosis by the AI system. The most frequently identified pathological findings were atrial fibrillation (4.3%), ST-segment changes (4.0%), and right bundle branch block (4.0%). Less common diagnoses included left bundle branch block (2.5%), first-degree AV block (1.5%), and sinus bradycardia (0.6%). A small number of patients (<1%) had combinations of abnormalities, such as bundle branch blocks with bradycardia or ST changes. Agreement between AI-based and manual ECG interpretations varied across diagnostic categories (*Table 8*). Moderate agreement was observed for LBBB (*κ* = 0.518) and RBBB (*κ* = 0.451), both with high specificity (>99%) and PPV (>78%). Atrial fibrillation showed fair agreement (*κ* = 0.403), with excellent specificity (99.8%) and PPV (96.6%), but low sensitivity (28.3%). AV block demonstrated poor agreement (*κ* = 0.083) and very low sensitivity (6.2%). Sinus bradycardia and tachycardia yielded low kappa values (0.280 and 0.161) and sensitivities below 30%, despite high specificities. The ‘None’ category showed moderate sensitivity (93.6%) but low specificity (36.2%) and a kappa of 0.338.

**Table 8:** Agreement between AI-based and manually assigned ECG diagnoses

Diagnostic category	Cohen’s Kappa	Agreement (%)	Sensitivity (%)	Specificity (%)	PPV (%)	NPV (%)	Accuracy	AUC
Atrial fibrillation	0.403	89.7	28.3	99.8	96.6	89.1	89.4	0.641
RBBB	0.451	36.7	66.7	99.0	78.6	98.3	97.4	0.829
1° AV Block	0.083	66.7	6.20	99.3	66.7	81.9	81.6	0.527
LBBB	0.518	45.0	51.7	99.4	78.9	97.8	97.3	0.755
Sinus bradycardia	0.280	50.0	16.7	99.7	50.0	98.5	98.2	0.582
Sinus tachycardia	0.161	10.0	26.9	96.5	23.3	97.1	93.8	0.617
None	0.338	70.2	93.6	36.2	70.4	77.7	71.7	0.649

Agreement calculated as percentage of cases where AI-based and manually assigned diagnoses matched. Sensitivity, specificity, PPV, and NPV calculated with manually assigned diagnoses as reference standard.

PPV, positive predictive value; NPV, negative predictive value; LBBB, left bundle branch block; RBBB, Right bundle branch block; AV, atrioventricular; CI, confidence interval; AUC, area under the curve.

## Discussion

This study evaluated the performance of three different AI-based ECG analysis tools in patients diagnosed with HCM. Our findings provide important information about the capabilities and limitations of these tools when applied to a specific heart disease population, rather than the general or mixed populations in which they were originally developed and validated. The AI tools evaluated in our study have undergone a comprehensive development and validation process on large datasets that explicitly include healthy controls and heterogeneous populations, and have reported high discrimination criteria in their original publications. Accordingly, our aim was not to replicate or revalidate these diagnostic performance results. Instead, our study was designed as a post-validation, disease-focused assessment aimed at characterizing how these AI tools perform in a cohort of validated HCM patients. Specifically, we aimed to examine probability distributions across the clinical spectrum of HCM, agreement with expert ECG interpretation, and correlations with ECG abnormalities and disease severity. Our study includes the analysis of image-based and single-centre algorithms, and these findings should not be interpreted as a general assessment of AI-based ECG analysis.

### HCM probability tool performance

Perhaps the most striking finding of our study was the relatively uniform distribution of AI-calculated HCM probabilities across our cohort, despite all patients having a confirmed diagnosis of HCM. The median probability was only 38.8% (IQR: 12.8–63.4%), considerably lower than expected for a tool specifically designed to detect HCM. This performance raises several important considerations.

First, this finding highlights the challenges of applying AI tools developed in general or mixed populations to specific disease cohorts. The HCM Probability Tool was likely trained and validated on datasets where HCM cases represented a small minority among various other cardiac conditions and normal individuals.^6^ When applied to an exclusively HCM population, the tool appears to lose discriminative power, possibly due to the absence of the contrast provided by non-HCM cases in the training data. Second, the dramatic difference in HCM probabilities between patients with normal vs. abnormal ECGs [40.5% (IQR: 13.8–63.7%) vs. 1.92% (IQR: 1.75–2.68%), *P* < 0.001] suggests that the AI algorithm heavily relies on overt ECG abnormalities for HCM detection. This is problematic given that a subset of HCM patients may have normal or near-normal ECGs, as previously reported in the literature.^2,10^ Image-based ECG analysis has shown poor performance (mean probability <2%) in these patients and may lead to inadequate identification, which could limit the algorithm's use for screening or early diagnosis purposes. Third, the correlation between HCM probabilities and markers of disease severity (maximal wall thickness *r* = 0.30, NT-proBNP *r* = 0.41, left atrial diameter *r* = 0.16) indicates that the tool may be more effective at identifying advanced or more phenotypically expressed HCM rather than subtle or early disease. This limitation could reduce its utility for early detection or screening purposes, where identifying less obvious cases is particularly valuable.^11,12^ Fourth, the significant variation in HCM probabilities across different HCM types, with apical variant showing the highest probabilities [58.0% (30.3–71.9%)] compared with obstructive [38.0% (10.4–63.2%)] and non-obstructive [36.6% (14.1–60.6%)] variants, suggests that the algorithm may be more sensitive to certain HCM phenotypes. This phenotype-specific performance variation could impact the tool’s clinical utility across the spectrum of HCM presentations.

### Structural heart disease probability tool performance

The SHD Probability Tool demonstrated better overall performance in our HCM cohort, with a median probability of 51.4% [IQR: 28.7–74.5%]. This improved performance likely reflects the broader scope of this tool, which was designed to detect various structural abnormalities rather than a specific condition like HCM.^7,13^ The correlations between SHD probabilities and parameters such as NT-proBNP (*r* = 0.38), QTc interval (*r* = 0.31), and QRS duration (*r* = 0.33) suggest that this tool may be capturing a wider range of cardiac abnormalities commonly associated with HCM. The negative correlations with LVEF by echocardiography (*r* = −0.22) and CMR (*r* = −0.16) further indicate sensitivity to functional consequences of SHD. However, similar to the HCM Probability Tool, the SHD tool showed significantly lower probabilities in patients with normal ECGs [10.1% (IQR: 5.66–15.2%) vs. 52.3% (IQR: 29.3–75.2%), *P* < 0.001]. This consistent pattern across both tools reinforces the notion that current AI algorithms may have limited sensitivity for detecting cardiac abnormalities in the absence of ECG changes, potentially missing cases where structural abnormalities precede electrical manifestations.^8,14,15^

### Implications for clinical application

Our findings have important implications for the clinical application of AI-based ECG analysis tools. The stark contrast in performance between patients with normal vs. abnormal ECGs suggests that these tools may have significant blind spots, particularly for HCM patients without characteristic ECG changes.^2,16^ This limitation is especially concerning given that early detection of HCM, often before the development of obvious ECG abnormalities, is a key goal in clinical practice.^17,18^ The variation in performance across different HCM phenotypes (apical, obstructive, non-obstructive) further complicates the clinical application of these tools. If AI algorithms are more sensitive to certain HCM variants than others, this could lead to inconsistent detection rates and potentially biased clinical decision-making. The associations between AI-calculated probabilities and markers of disease severity suggest that these tools may be more useful for risk stratification than for initial diagnosis.^19,20^ The significant correlations with parameters like NT-proBNP, maximal wall thickness, and left atrial diameter indicate that AI-derived probabilities might provide insights into disease progression and severity, even if their diagnostic performance is suboptimal.^12,19^

### Comparison with previous literature

Our findings both complement and contrast with previous studies evaluating AI-based ECG analysis tools. The original validation studies for these tools reported substantially higher performance metrics in their respective test populations.^3,6,7^ For instance, previous studies on HCM detection using AI-enabled ECG analysis reported area under the receiver operating characteristic curve values exceeding 0.90, considerably higher than what our distribution of probabilities would suggest.^15,18^ This discrepancy underscores an important limitation of current AI approaches: performance metrics derived from general or mixed populations may not translate directly to specific disease cohorts. This phenomenon, sometimes referred to as ‘spectrum bias’ in diagnostic testing literature, occurs when test performance varies across different patient subgroups or disease spectrums.^21^ Previous studies by Attia *et al*. and Ko *et al*. on AI-based ECG analysis for detecting cardiac conditions have similarly noted reduced performance when algorithms are applied to populations different from their training data.^8,13^ Our results extend these observations specifically to HCM patients, highlighting the need for disease-specific validation before clinical implementation.

### Strengths and limitations

Our study has several strengths, including the large, well-characterized HCM cohort (*n* = 681), comprehensive clinical and imaging data, and systematic evaluation of multiple AI tools within the same population. The inclusion of correlation analyses with various clinical and imaging parameters provides valuable insights into the factors influencing AI tool performance.

However, several limitations should be acknowledged. First, our study was conducted at a single centre with a relatively homogeneous patient population, potentially limiting generalizability. Second, we did not have access to the training data or internal algorithms of the AI tools, preventing detailed analysis of the specific features driving their predictions. Third, the cross-sectional nature of our study precludes assessment of how these tools might perform in longitudinal monitoring of HCM patients. Fourth several high-performing HCM AI algorithms reported in the literature utilize raw digital ECG signals rather than digitized ECG images. Image-based ECG analysis introduces potential confounders related to image resolution, contrast, grid detection, and signal distortion, which are largely absent in raw digital ECG files. Finally, we did not include a control group of non-HCM patients, which would have allowed for calculation of traditional diagnostic performance metrics such as sensitivity and specificity for HCM detection. Despite these limitations, our findings provide important insights into the current capabilities and limitations of AI-based ECG analysis tools in HCM patients, with implications for their potential clinical application in this specific population.

## Conclusion

While AI-based ECG analysis tools show potential for enhancing cardiac care, their application in specific disease populations like HCM requires careful consideration of their limitations. Continued refinement and validation of these tools in diverse and disease-specific cohorts will be essential for realizing their full potential in clinical practice.

## Data Availability

The data underlying this article will be shared on reasonable request to the corresponding author.
